# Lipid Production from Native Oleaginous Yeasts Isolated from Southern Chilean Soil Cultivated in Industrial Vinasse Residues

**DOI:** 10.3390/microorganisms11102516

**Published:** 2023-10-09

**Authors:** Paola Díaz-Navarrete, Luis Marileo, Hugo Madrid, Carlos Belezaca-Pinargote, Patricio Dantagnan

**Affiliations:** 1 Departamento de Ciencias Veterinarias y Salud Pública, Facultad de Recursos Naturales, Universidad Católica de Temuco, Temuco 4780000, Chile; 2 Núcleo de Investigación en Producción Alimentaria, Departamento de Ciencias Agropecuarias y Acuícolas, Facultad de Recursos Naturales, Universidad Católica de Temuco, Temuco 4780000, Chile; 3 Programa de Doctorado en Ciencias Agropecuarias, Facultad de Recursos Naturales, Universidad Católica de Temuco, Temuco 4780000, Chile; lmarileo@uct.cl; 4 Departamento de Tecnología Médica, Facultad de Ciencias de la Salud, Universidad de Tarapacá, Iquique 1101783, Chile; hugo.madrid@gmail.com; 5 Facultad de Ciencias Agrarias y Forestales, Universidad Técnica Estatal de Quevedo, Quevedo 120501, Ecuador; cbelezaca@uteq.edu.ec

**Keywords:** microbial lipids, oleaginous yeasts, triglycerides (TGs), vinasse residual

## Abstract

In this research, six strains of oleaginous yeasts native to southern Chile were analyzed for their biotechnological potential in lipid accumulation. For this purpose, the six strains, named PP1, PP4, PR4, PR10, PR27 and PR29, were cultivated in a nitrogen-deficient synthetic mineral medium (SMM). Then, two strains were selected and cultivated in an industrial residual “vinasse”, under different conditions of temperature (°C), pH and carbon/nitrogen (C/N) ratio. Finally, under optimized conditions, the growth kinetics and determination of the lipid profile were evaluated. The results of growth in the SMM indicate that yeasts PP1 and PR27 presented biomass concentrations and lipid accumulation percentages of 2.73 and 4.3 g/L of biomass and 36.6% and 45.3% lipids, respectively. Subsequently, for both strains, when cultured in the residual vinasse under optimized environmental conditions, biomass concentrations of 14.8 ± 1.51 g/L (C/N 80) and 15.83 ± 0.57 g/L (C/N 50) and lipid accumulations of 28% and 30% were obtained for PP1 and PR27, respectively. The composition of the triglycerides (TGs), obtained in the culture of the yeasts in a 2 L reactor, presented 64.25% of saturated fatty acids for strain PR27 and 47.18% for strain PP1. The saturated fatty acid compositions in both strains are mainly constituted of fatty acids, myristic C 14:0, heptadecanoic C 17:0, palmitic C 16:0 and stearic C 18:0, and the monounsaturated fatty acids constituted of oleic acid C 18:1 (cis 9) (28–46%), and in smaller amounts, palmitoleic acid and heptadecenoic acid. This work demonstrates that the native yeast strains PP1 and PR27 are promising strains for the production of microbial oils similar to conventional vegetable oils. The potential applications in the energy or food industries, such as aquaculture, are conceivable.

## 1. Introduction

Oleaginous yeasts, together with microalgae and bacteria, have been presented as an alternative oil-producing source to those of conventional marine or terrestrial plant origin [1,2,3]. Under appropriate culture conditions, these microorganisms store a greater percentage of lipids (25–70% or more) in their cellular composition, with a lipid profile consisting of long fatty acid chains and a high degree of saturation [e.g., palmitoleic (16:1), oleic (18:1) and myristic (14:0) acids, among others] comparable to conventional vegetable oils (rapeseed, soybean and jatropha) currently used in different types of industries [1,4]. Yeasts have been referred to as organisms with the greatest biotechnological potential due to their ability to adapt to various environments, their cell structure with a variety of primary and secondary metabolites of great industrial interest, and a relatively high growth rate, among other characteristics. The production of numerous metabolites by yeasts grown on agricultural, industrial, and food residues and wastes is a highly viable alternative for the chemical and food industries. In addition, this production helps reduce environmental pollution due to the conversion of waste substrates and by-products into value-added products [5,6,7]. Moreover, there are advanced standardized technologies for their cultivation, they do not depend on the availability of arable land and have a low water and carbon footprint [8,9].

Considering these advantageous aspects, yeasts are presented as potential lipid sources for various industries that require these nutrients [1,10]. For instance, fish production, and especially salmon production, is one of the most demanding users of dietary lipids in animal production. The lipids used are mainly fish and vegetable oils. The production of these oils has been questioned environmentally and is not viable over time, considering the projected demand for their use in the coming years [1,11]. For this reason, there has been increasing interest in discovering sustainable and cost-effective new alternative lipid sources for use in the diet of cultured aquatic animals [11,12]. 

Several authors have reported lipid accumulation in yeasts [11,13,14] with the most promising species for fat production being the following: *Rhodosporidium toruloides*, *Lipomyces starkeyi*, *Lipomyces tetrasporus*, *Cutaneotrichosporon curvatum*, *Candida diddensiae*, *Metschnikowia reukaufii*, *Candida tropicalis*, *Solicoccosyma terricola*, *Naganishia albida*, *Papiliotrema laurentii*, *Rhodotorula glutinis*, *Rhodotorula graminis*, *Rhodotorula mucilaginosa*, *Apiotrichum domesticum*, *Trichosporon asahii*, *Tausonia pullulans*, *Yarrowia lipolytica* and *Schwanniomyces etchellsii* [15,16,17,18,19]. Most of these yeasts accumulate lipids mainly in the cytoplasmic membrane (about 25% of the cell dry weight) in the form of triglycerides (TGs) [20], noting that the major production of these TGs is under conditions of nutrient limitation such as nitrogen, phosphorus and an excess carbon source [21,22,23]. 

Research regarding the factors that influence the accumulation of lipids and the profile of fatty acids in yeasts and other oleaginous microorganisms is mainly focused on (i) the optimization of lipid production through stress by exogenous factors [24]; (ii) the inclusion of raw materials issuing from industrial waste [25,26]; (iii) the search for culture strategies (batch, fed-batch and continuous cultures) [27,28,29,30]; and (iv) the determination of metabolic and molecular aspects involved in lipid accumulation [2,31]. 

Concerning stress due to exogenous factors, the influential variables in the accumulation of lipids are temperature, an increase in osmotic pressure (high concentrations of sodium chloride and sugars), nitrogen source starvation, modification of the carbon/nitrogen (C/N) ratio, the presence of toxic substances (e.g., heavy metal salts, phenol and methylene blue), exposure to visible or ultraviolet radiation, and the presence of oxidative stress [18,29,30,31,32]. In response to these unfavorable environmental conditions, microbial cells show an increased expression of (specific) genes for the production of enzymes that are involved in lipid biosynthesis [16,33].

In terms of the inclusion of waste-based raw materials, the identification of residues with the potential to be used as a substrate (carbon source) for the cultivation of oleaginous yeasts is essential to industrialize the bioprocess [34,35]. It has also been reported that culture strategies make it possible to optimize cell biomass production and, therefore, the concentration of primary and secondary metabolites [36]. Various authors indicate that a culture in two stages is essential for greater lipid productivity [36,37,38,39]. In the first stage, emphasis is placed on the accumulation of microbial biomass (with a relatively low C/N ratio and rich in nutrients), and in the second stage, the amount of lipids is increased using, for example, solutions with a high concentration of carbon sources (with a relatively high C/N ratio and nitrogen limitation) [40,41,42]. Another of the strategies to increase lipid productivity is the study of metabolic and molecular pathways [3,43]. In this regard, researchers such as Tang et al. [43] conducted a study on the gene encoding enzyme isocitrate dehydrogenase in *L. starkeyi* and discovered that enzymatic activity decreased upon the onset of lipid accumulation. Similarly, authors Gemperlein et al. [3] carried out the design and construction of an artificial PUFA (polyunsaturated fatty acid) biosynthetic gene set. After performing the gene integration, they cultivated *Y. lipolytica* under conditions of phosphorus deprivation and obtained 16% DHA (docosahexaenoic acid). From the standpoint of metabolic studies, authors Xue et al. [44], using metabolic engineering in *Y. lipolytica*, were able to enhance the accumulation of eicosapentaenoic acid (EPA) from 15% to 56.6% in respect of the dry weight of the cell.

It is important to note that the composition of fatty acids with different carbon chain lengths, with either an even or odd number of carbons, has significantly different characteristics and wide applications in energy, materials, medicine and nutrition [41]. In the energy field, it is known that there are yeasts with lipid profiles similar to those needed to make biodiesel [21]. Similarly, there are some strains described as *S. cerevisiae*, in which, incorporating phytomics and multiomics technologies, food products similar to existing ones could be generated, for example, in the case of cocoa butter, where a decrease in the availability of vegetable raw materials for its production is projected [45]. Another area in which the use of unicellular oils from yeasts has begun to be examined is aquaculture, suggesting that several yeasts may have lipid profiles similar to the conventional oils currently used in the production of fish feed [29,46]. An alternative route to meeting the future demand for oils could be to utilize oleaginous yeasts as microbial cell factories for the sustainable production of microbial lipids [47,48]. The aim of the present work is to evaluate the lipid accumulation capacity of native yeasts from southern Chile cultivated in an industrial “vinasse” residue.

## 2. Materials and Methods

### 2.1. Yeast and Culture Conditions

Six yeast strains named PP1, PP4, PR4, PR10, PR27 and PR29 were included in this study. These strains were isolated from soil of the Los Ríos region, Chile, and showed biotechnological potential for lipid accumulation in a previous study by Díaz et al. [49]. The strains were maintained in sabouraud agar slants at 4 °C with subcultures being performed every three months. Likewise, a constant stock of Petri dishes with Potato Dextrose Agar was maintained at 4 °C and replicated every 15 days. 

### 2.2. Industrial Residual Substrate Vinasse (IRV) P5

The complex culture medium used in the present research was the industrial residue vinasse (IRV) generated by a local company producing baker’s yeast called “Collico”, located in the city of Valdivia, Chile (40° S). Table 1 shows the physicochemical characterization of the residue.

### 2.3. Evaluation of Lipid Accumulation of the Six Yeast Strains in Synthetic Mineral Medium (SMM) and Selection of Two Oleaginous Strains

Six yeast strains were initially evaluated, these were incubated in a synthetic mineral medium (SMM), which was nitrogen limited and had an excess carbon source. This medium contained the following composition: KH_2_PO_4_, 0.7%; Na_2_HPO_4_, 0.25%; MgSO_4_·7H_2_O, 0.15%; CaCl_2_, 0.015%; FeCl_3_·7H_2_O, 0.015%; ZnSO_4_·7H_2_O, 0.002%; (NH_4_)_2_SO_4_, 1%%; yeast extract, 0.05%; and glucose, 6.0%; adjusted to pH 6.0 [50]. Previously, the yeast strains were inoculated in 100 mL of SMM for 36 h at 150 rpm and 25 °C (preculture). This pre-culture, adjusted to 10^6^ cells/mL, was suspended at 5% (*v*/*v*) in 200 mL of SMM and incubated at 200 rpm at 25 °C for 6 days. After that time, it was centrifuged at 5000 rpm for 20 min and washed three times with distilled water. Biomass was determined by weighing on an analytical balance and it is expressed as cell dry weight (g/L) according to Sluiter et al. [51], and lipid determination was carried out using the Folch method [52]. From these data, the two best yeast strains were selected. The selection criterion was according to the highest percentage of lipids obtained in each strain. The control strain used in the experiments was *Rhodotorula glutinis* ATCC 16725 (RHO), known as a typical oleaginous yeast [53].

### 2.4. Effect of Physicochemical Variables on Lipid Accumulation in Selected Strains

Once the two yeast strains with the highest lipid accumulation potential were selected, the effect on cell growth and lipid accumulation in IRV was determined by evaluating different physicochemical variables such as T° (25, 30 and 37 °C), C/N ratio 20, 50, 80 and pH (4, 5.5 and 7). For each experiment, 200 mL of autoclaved vinasse was prepared at 121 °C for 15 min. Each flask was inoculated with 10^6^ cells/mL, at 5% (*v*/*v*). The experiments were performed in triplicate and were incubated for 5 days at 150 rpm. At the end, g/L dry weight and total lipid concentration were determined. From these results, the culture variables favoring lipid accumulation and g/L biomass in each yeast strain were selected. To adjust the C/N ratio, glucose was considered to contribute 0.4 g C/g of glucose, and NaOH and HCl 1 N were used to adjust the pH. 

### 2.5. Kinetics of Growth and Lipid Accumulation in Optimized Industrial Residual

These experiments were conducted in triplicate in 1 L Erlenmeyer flasks with a usable volume of 400 mL of IRV. Each flask was inoculated with an initial suspension of 10^6^ cells/mL of a 5% (*v*/*v*) exponential phase pre-culture of PR27, PP1 and RHO as appropriate, and incubated at 200 rpm and 25 °C, for 6 days with constant aeration of 2 vvm. The trials were performed under improved IRV conditions obtained previously. Samples of 10 mL were taken every 12 h to determine growth kinetics [($\ln\left(X\right)=\ln(X_{0})+\mu *t$, Y_X_ = Cell yield (grams of yeast produced/grams of glucose consumed), lipid productivity = grams of lipids/per grams biomass liter of vinasse per day)].

### 2.6. Analytical Methods

#### 2.6.1. Quantification of Cell Biomass (Yeast) and Lipids

The post-culture yield of yeast cell biomass was determined after centrifugation in a previously weighed thimble (3000× *g*, 10 min and 4 °C). The centrifuged cell biomass was dried at a temperature of 80 °C (WTC binder oven) for 24 h to achieve a constant weight. The biomass yield was expressed in grams of yeast dry weight (g d.w.) per liter of culture medium (g d.w.L^−1^ of medium) [54]. Total lipids were extracted according to Guerreiro et al. [55], taking 1 g of sample and using a chloroform:methanol solution (2:1 and 0.01% BHT) as extract, in addition to 15 mL of HCL 0.1 N and 10 mL of MgCl 0.5%. This solution was homogenized in vortex, centrifuged at 3500 rpm, and the chloroform phase was brought to dryness and constant weight under nitrogen stream. The value obtained was expressed as a percentage of total lipids.

To obtain the fatty acid methyl ester, the total lipids were methylated according to the method of Morrison and Smith [56]. The methyl esters were then ready to be injected (1 µL), using an Agilent 7683B Series (Wilmington, NC, USA) automatic injector attached to a Hewlett Packard 6890 series II Plus (Wilmington, NC, USA) chromatograph equipped with a flame ionization detector (FID), with He as carrier gas. The separation was performed through the (Supelco, Bellefonte, PA, USA) capillary column (30 m length × 0.25 internal diameter × 0.20 µm film thickness). The injector and detector temperature were 220 and 200 °C, respectively. To ensure the best possible separation of the fatty acids, the following programming was used, 60 °C for 1 min, followed by 4 °C per minute at 204 °C, and, finally, 2 °C per minute at 220 °C and stabilization for 2 min. Fatty acids were identified by comparison with Supelco-37 standard fatty acids (Sigma-Aldrich, Darmstadt, Germany) and quantified in HPCHEM Stations software (Agilent Technologies, Santa Clara, CA, USA), and expressed as percent area according to the total fatty acids identified. Reducing sugars were determined following the DNS methodology described by Sluiter et al. [51].

#### 2.6.2. PCR Amplification and Yeast DNA Sequencing

The isolated yeasts were cultured in YEPD for 30 h at 25 °C, then the DNA of the yeast strains was extracted using the yeast DNA EZNA kit using the standard protocol. The nuclear ribosomal DNA (rDNA) region encompassing the internal transcribed spacer 1, 5.8S RNA gene, and internal transcribed spacer 2 (ITS1-5.8S-ITS2 and part of the 28S gene fragment were amplified and sequenced using primers ITS1 (5′ TCC GTA GGT GAA CCT GCG G 3′) and NL4 (5′ GGT CCG TGT TTC AAG ACG G 3′) [57,58]. Amplification was performed in 25 μL of reaction composed of GoTaq^®^ Green Master Mix (Taq DNA polymerase, dNTPs, MgCl_2_, Promega) and 0.5 μL of forward and reverse primer to amplify 200 ng of template DNA. The conditions for the amplification reaction were as follows: initial denaturation (2 min at 95 °C), final denaturation (1 min at 94 °C), annealing (40 s at 54 °C), extension (1 min at 72 °C) and final extension (10 min at 72 °C). The PCR reaction was performed for 30 cycles. A total of 8 μL of the PCR product was analyzed at 1.5% agarose in 1X TAE, then visualized and photographed on a UV transluminator (PHOTO/UV 21). Amplicon size was determined using the 100-bp DNA molecular weight marker (Thermo scientific). DNA concentration was measured on a NanoDrop 1000 spectrophotometer (Infinite M200). For sequencing, the PCR-amplified DNA was purified with the EZNA extraction kit, following the standard protocol. The purified product was sent to MACROGEN, KOREA and sequenced with the same primers used in amplification.

#### 2.6.3. Molecular identification of yeast strains

In this study, the assembly of nucleotides were analyzed more clearly using BioEdit. BLAST searches were carried out in order to compare the sequences of the studied strains with those of species currently represented in GenBank. ITS sequences of closely related taxa, especially ex-type strains or other reliably labeled strains, were retrieved for phylogenetic analyses. The phylogenetic studies were based on the ITS locus because it provides a higher resolution than LSU in yeasts [57] and because the latter locus was not available for some reference strains in GenBank. DNA sequence alignments were made with the MUSCLE webserver (http://www.ebi.ac.uk/Tools/msa/muscle/, Edgar 2004, accessed 22 September 2023) and then adjusted manually with a text editor. Phylogeny reconstructions were performed with the maxi mum likelihood and neighbor-joining methods with MEGA XI [58], using the best DNA substitution models chosen by that software. The statistical support for the groupings was assessed using bootstrap analysis of 1000 replicates. 

### 2.7. Statistical Analysis

Statistical analyses were performed using the R program. Data were analyzed using one-way analysis of variance (ANOVA) and the Student’s *t* test, as appropriate. Differences observed at *p* ≤ 0.05 were considered significant. Pearson’s correlation was performed using the SPSS software version 26 (IBM, Armonk, NY, USA).

## 3. Results

### 3.1. Molecular Identification of Strains

**Figure 1:**
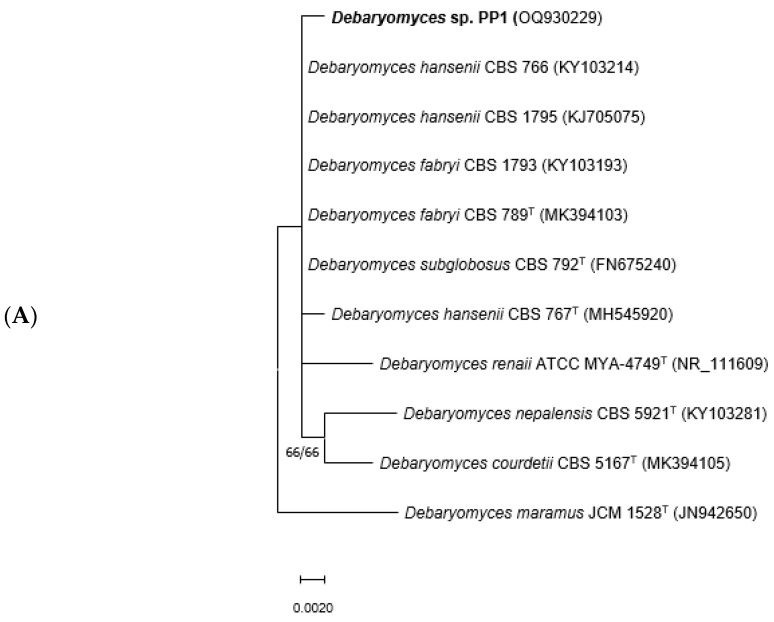

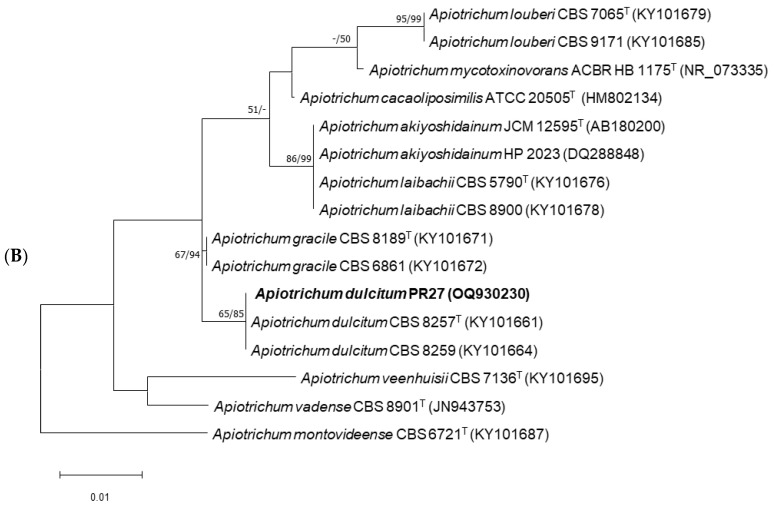
Maximum likelihood (ML) trees constructed with the ITS sequences of strains PP1 and PR27 and related species of *Debaryomyces* (**A**) and *Apiotrichum* (**B**), respectively. Branch lengths are proportional to distance. Bootstrap values ≥50% obtained in ML and neighbor-joining analyses, respectively, are shown near the internodes. Isolates studied in this work appear in bold type. T, ex-type strain. GenBank accession numbers of ITS sequences are given in parenthesis after strain numbers.

In BLAST searches with the ITS/LSU sequence of strain PP1, the closest matches were *Debaryomyces fabryi* CBS 789, ex-type strain (GenBank MK394103, identities = 1098/1103 (99.55%), two gaps), *D. hansenii* CBS 767, ex-type strain (GenBank MH545920, identities 1097/1103 (99.46%), two gaps), *D. subglobosus* CBS 792, ex-type strain (GenBank FN675240, identities 544/547 (99.45%), 1 gap), and other strains labeled as these species. These taxa are phylogenetically very closely related and constitute the “*Debaryomyces hansenii* species group” [59]. In a phylogenetic tree based on ITS sequences of members of that species group and related taxa (Figure 1A), *D. hansenii, D. fabryi* and *D. subglobosus* could not be distinguished properly due to a lack of resolution, and the species-level identity of strain PP1 was uncertain. This fungus was, therefore, identified as “*Debaryomyces* sp.”. The closest matches of strain PR27 in BLAST searches with its ITS/LSU sequence were *Apiotrichum dulcitum* strain SEG-8-30 (GenBank FR716597, identities = 1020/1026 (99.42%), 3 gaps), *A. cacaoliposimilis* ATCC 20505, ex-type strain (GenBank HM802134, identities = 1008/1025 (98.34%), 2 gaps), *A. akiyoshidainum* JCM 12595, ex-type strain, (GenBank AB180200, identities = 1000/1026 (97.47%), 3 gaps), and other strains of these species. Based on these results, strain PR27 was preliminarily identified as *A. dulcitum*. This identification was confirmed in a phylogeny reconstruction based on ITS sequences of several *Apiotrichum* species (Figure 1B), where strain PR27 grouped with the ex-type strain of *A. dulcitum*, CBS 8257, and with another reference strain of that species, CBS 8259. 

### 3.2. Yeast Culture in Synthetic Mineral Medium (SMM)

**Figure 2:**
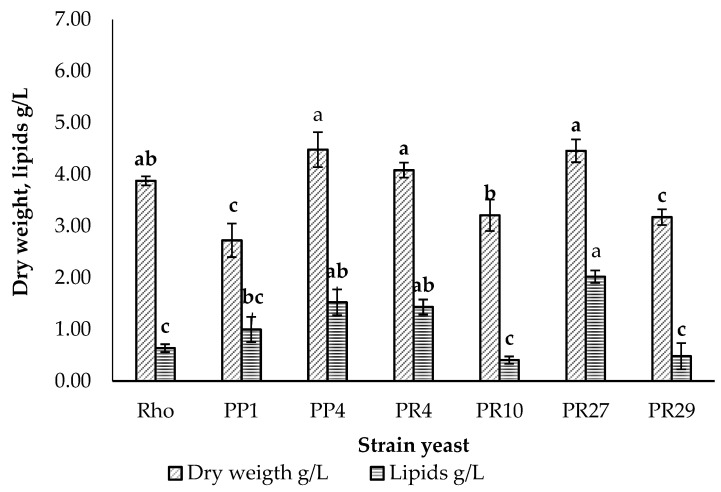
Cell growth and lipid accumulation after 5 days of culture in yeast strains. Different letters indicate significant differences.

The six strains were cultured with glucose as the carbon source and stressed by decreasing the concentration of the nitrogen source. Figure 2 shows the results obtained, where strains PP4 and PR27 are the ones that presented the highest biomass values, 4.48± and 4.04± g/L, respectively. However, when analyzing the percentage of total lipids, it can be seen that strain PP1 reached a value of 36.6% and PR27 a value of 45.3%, unlike strains PP4 and PR4, which were around 35% and were very similar to the control strain (RHO) that reached 35.9%; a very different situation is seen in strains PR10 and PR29, where the percentage of accumulation with respect to dry weight did not exceed 20% in all cases. 

### 3.3. Effect of Physicochemical Variables on Lipid Accumulation in Selected Strains Cultured in IRV

The vinasse used in this research has a soluble carbohydrate content of 8%, total protein content of 2.6% and 10° Brix. According to Table 2, in strain PP1, differences are observed for cell growth with a temperature of 25 °C, notably favoring the cell growth rate. Increasing the temperature to 30 °C reduced the cell biomass from 7.52 g/L to 6.35 g/L, and at 37 °C, the biomass reached a value of 6.05 g/L, being lower than for the temperatures previously analyzed. The temperature that favored the highest concentration of lipids was 30 °C, reaching a value of 1.95 g of lipids/L of vinasse.

With respect to pH, the value that favored cell growth to the detriment of lipid accumulation was pH 4, where a yield of 13.86 g/L of biomass and 1.72 g/L of lipids, respectively, was achieved. The highest concentration of total lipids was reached at pH 7, where 1.97 g of lipids/L of vinasse was obtained. The C/N ratio that favored cell growth was 50, reaching 15.83± g of yeast/L of vinasse with 2.29 ± 0.782 g of lipids/g of yeast. At the C/N ratio = 80, 4.614 ± 0.148 g of lipids/g of yeast was obtained.

With respect to strain PR27, it was observed that, at 25 °C, the cell growth rate was favored, obtaining 5.21 g/L of biomass and 2.81 g of lipids/L vinasse. Increasing the temperature to 30 °C reduced the cell biomass to 4.28± g/L, and at 37 °C the biomass reached a similar value of 4.2± g/L. The pH value that favored cell growth to the detriment of lipid accumulation was pH 5.5, where 7.68 g/L of biomass (dry weight) and 1.06 g/L of lipids were obtained. This last variable was favored at pH 7 where 2.63 g/L of lipids were obtained. The C/N ratio that favored cell growth and lipid accumulation was 80, reaching 14.8 g/L of biomass and 4.68 g/L of lipids.

Pearson’s correlation was used to determine a significant correlation between the variables and the parameters evaluated. Table 3 shows that for strain PP1, there is a direct significant correlation (*p* < 0.05) between the biomass obtained with the C/N ratio (r = 0.876), in addition to a significant direct correlation between the total lipid content and the C/N ratio (r = 0.833). For this strain, a significant inverse correlation is observed between the temperature (r = −0.301) and the biomass obtained. For the biomass obtained with strain PR27, there is a significant inverse correlation with temperature (r = −0.413) and a direct correlation with the C/N ratio (r = 0.895). The total lipid content for this strain is directly correlated with the C/N ratio (r = 0.655) and pH (r = 0.586), while there is a significant inverse correlation with temperature (r = −0.406).

### 3.4. Determination of Growth Kinetics of Yeasts Cultured in Vinasse

**Figure 3:**
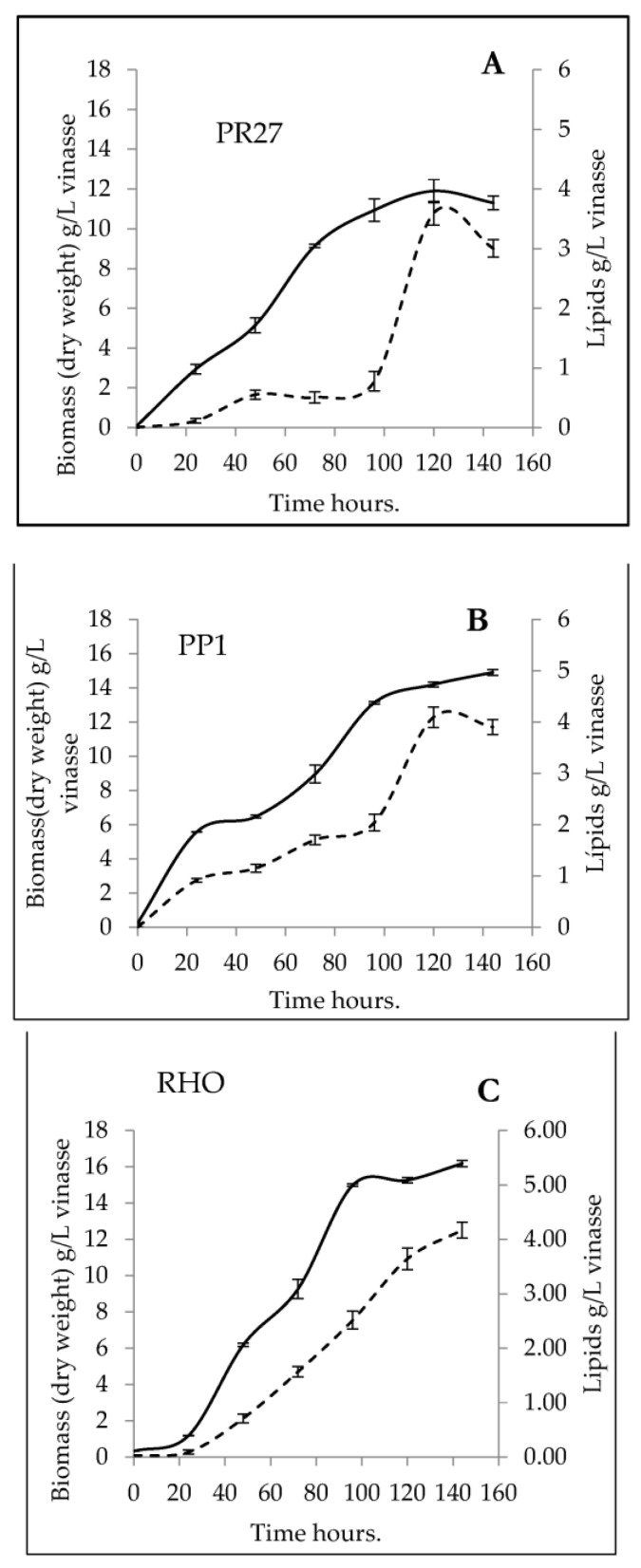
Cell growth and lipid content of yeast strains. (**A**) = PP1, (**B**) = PR27, (**C**) = RHO cultured in vinasse. Black line: dry weight (g yeast/L vinasse); dashed line: lipid content (g lipids/L vinasse).

As a result of 144 h of culture, for the experiments using the industrial residual vinasse as the culture medium, it can be seen that there is a definition of the cell growth stages. It is observed in Figure 3A,B (PP1 and PR27) that the lag phase in both yeasts occurs in less than 24 h. The exponential growth stage for PP1 culminated at 96 h with a maximum of 13.13 g of yeast/L vinasse, unlike PR27, which was established at 120 h with 11.89 g of yeast/L vinasse. In RHO, there is a lag phase during the first 24 h. After this time, the exponential phase of growth clearly begins, reaching the maximum biomass at 96 h with a value of 14.9 g of yeast/L vinasse. The PP1 yeast, after 120 h, produced 4.09 g of lipids/L vinasse. For yeast, PR27, after 120 h, reached a content of 3.59 g of lipids/L vinasse. Figure 3C for the yeast RHO, it can be seen that, up to 24 h, there is no increase in the lipid content. But after that time, the number of lipids in vinasse goes up and reaches 4.17 g of lipids/L vinasse after 144 h.

Table 4 shows the growth rate values for each of the yeasts selected in the present study. Strain PP1 presented the highest specific growth rate with a value of 0.1388 h^−1^ when cultured in the industrial residual vinasse. For PR27, a µ(h^−1^) of 0.11 h^−1^ was reached and for RHO it was 0.19 h^−1^. With respect to cell yields, RHO was the strain that presented the highest affinity with the substrate with 0.12 g biomass/g of glucose consumed. With respect to lipid productivity, the control strain presented the highest value of 0.7 g L^−1^ d^−1^. 

#### Lipid Profile

**Figure 4:**
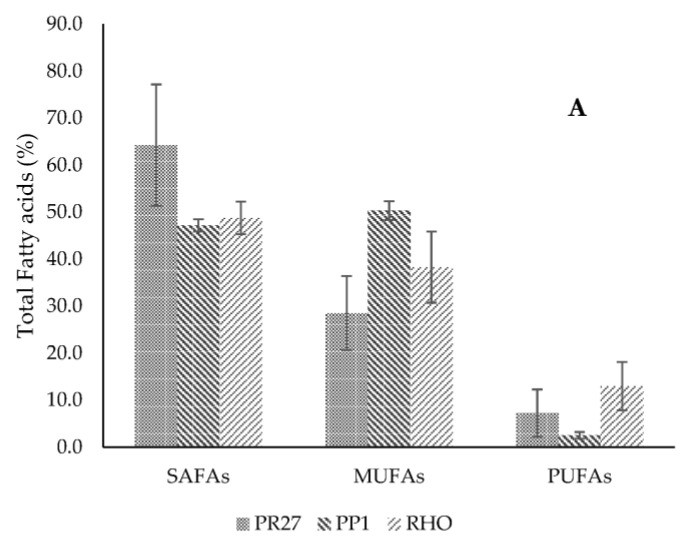

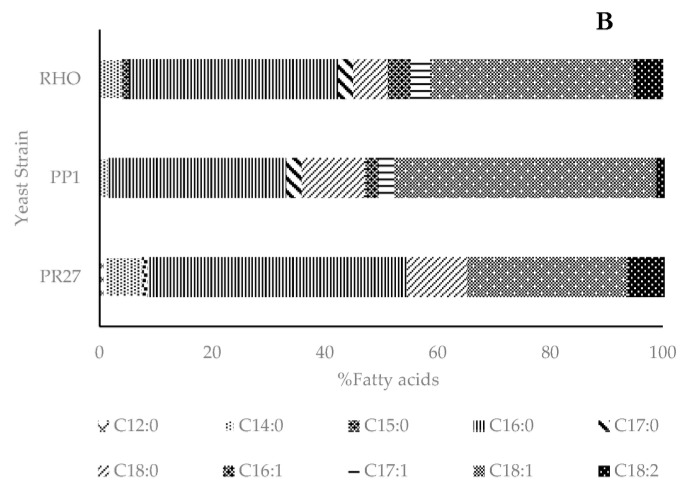
Percentage of fatty acids in PR27, PP1 and RHO strains (**A**); profile fatty acids of yeast strain (**B**).

The fatty acid profile for the yeasts studied has shown a high percentage of saturated and monounsaturated fatty acids, Figure 4A. For strain PR27, 64.25% of saturated fatty acids were obtained, whose composition is constituted of lauric acid (0,9%) C:12:0, myristic acid (6.7%) C 14:0, palmitic acid (45.9%) C 16:0 and stearic acid (11%) C 18:0 (Figure 4B). Monounsaturated fatty acids with 28.5% are entirely constituted of oleic acid (cis 9) C18:1 (28.5%), and polyunsaturated fatty acids are constituted of linoleic acid (cis-9 cis-12) C18:2 with 7.25%. For strain PP1, 47.18% of SAFA was obtained, whose composition is constituted of myristic acid C14:0 (1.55%), palmitic acid C16:0 (31.63%), heptadecanoic acid C17:0 (2.68%), and stearic acid C18:0 (11.33%) (Figure 4B). MUFAs correspond to 50.31% and are constituted of oleic acid (cis-9) C18:1 (46.54%), heptadecenoic acid C17:1 (2.76%) and palmitoleic acid C16:1 (2.4%). In the case of PUFAs, 2.52% of linoleic acid (cis-9 cis-12) C18:2 was obtained. With respect to the yeast RHO, 48.75% of SAFAs were obtained, whose composition is constituted of myristic acid C14:0 (4.66%), pentadecaenoic acid C15:0 (0.79%), palmitic acid C16:0 (34.83%), heptadecanoic acid C17:0 (2.44%) and stearic acid C18:0 (5.83). The accumulated MUFAs corresponded to 38.3% and are constituted of oleic acid (cis 9) C18:1 (30.83%), palmitoleic acid C16:1 (2.82%), and heptadecenoic acid C17:1 (3.4%). In the PUFAs, 6.37% of linoleic acid (cis-9 cis-12) C18:2 was accumulated. Supplementary material File S1.

## 4. Discussion

According to Silva et al. [60], the composition of vinasse can vary considerably depending on its origin. García and Rojas [61] reported that sugarcane vinasse contains a variety of plant nutrients, such as 18% oxidizable carbon, 5.9% total protein, and a series of organic acids and sugars, including 0.21% sucrose. The first two parameters are higher than those determined in the vinasse used for the yeast culture in our research. According to the previously cited works, vinasse composition will depend on the raw material that it originated from and the concentration of total solids it contains [62]. Thus, Díaz-Vásques et al. [25] used a residual tequila vinasse (TV) as a carbon source for the culture of the forage yeast species *Cyberlindnera jadinii, Kluyveromyces marxianus* and *R. mucilaginosa.* The results of that study revealed that TV is a substrate that allows the generation of up to 18.08 ± 2.73 g L^−1^ of high-protein yeast that could be used as a protein source for animal nutrition. Ayadi et al. [63] cultured *R. mucilaginosa* from lignocellulosic residues, in this case using wheat bran residues pretreated with diluted sulfuric acid, to make nutrients accessible to the yeast and start fermentation. Similar analyses are presented in a study by Xiaochen et al. [64], where lipid production was evaluated in five yeast strains—*Cutaneotrichosporon oleaginosum, R. glutinis, R. toruloides, L. starkeyi*, and *Y. lipolytica*—using a wheat straw hydrolysate treated with sulfuric acid. With the exception of *R. toruloides*, all yeasts were able to detoxify this medium and produce lipid accumulation. The yeast RHO evaluated in the present research reached 16.7 ± 1.11 g dry weight/L vinasse and 4.1 g total lipids/L vinasse. Another study conducted by Patel et al. [65] used lignocellulosic biomass as a substrate for yeast growth, allowing them to determine the effect of inhibitors on growth, metabolism and end products, such as 5-hydroxymethylfurfural (HMF), furfural, acetic acid and phenolic compounds. The residual vinasse was only sterilized, and no pretreatment was performed. With regard to the isolated yeast strains, DNA sequence comparisons and phylogenetic analyses showed that strains PP1 and PR27 represent *Debaryomyces* sp. and *Apiotrichum dulcitum*, respectively. Both genera have been described as capable of accumulating more than 25% of their dry cell weight in TG [49]. *Debaryomyces hansenii* is a non-conventional yeast considered to be a well-suited option for a number of different industrial bioprocesses. It possesses several beneficial traits, such as halotolerance, oleaginousness, xerotolerance and resistance to inhibitory compounds, which translate into several advantages for industrial fermentation facilities when compared to traditional microorganisms. The molecular mechanisms underlying this natural tolerance should be further researched to use this yeast broadly in biotechnological processes, even though it has been highly studied over the past three decades [59]. On the other hand, Arous et al. [17] analyzed the growth characteristics, lipid accumulation and composition during the life cycle of the yeast *Schwanniomyces etchellsii* (formerly *Debaryomyces etchellsii*) under nitrogen limitation conditions. This yeast, grown in batch flasks or bioreactor cultures, reproduced asexually by budding when nitrogen was available in the growth medium, or sexually by ascospores after nitrogen exhaustion, producing more than 7 g L^−1^ of biomass. During ascosporogenesis, there was a significant increase in the cellular lipid content in the dry cell mass, i.e., from a mass fraction of 11.9% in the vegetative phase to 22.4%. The yeast synthesized lipids containing long-chain fatty acids (mainly C16 and C18). In the present work, strain PP1 was able to accumulate 28% lipids in vinasse (Figure 3) with lipid profiles rich in saturated (47.18%) and monounsaturated (50.31%) fatty acids (Figure 4A).

The genus *Apiotrichum* has been revised to include new species, and there are about 27 species described [66,67]. It was decided to reinstate the genus *Apiotrichum* in order to accommodate species previously assigned to the brassicae/gracile and porosum clades of the genus *Trichosporon*. The phylogenetic reclassification of Tremellomycetes by Liu et al. [68] resulted in the placement of species previously classified in *Trichosporon* sensu lato into three genera, i.e., *Apiotrichum*, *Cutaneotrichosporon* and *Trichosporon* [69]. It has been reported that some species of *Apiotrichum* can accumulate lipids. These are *A. domesticum, A. dulcitum, A. curvatum, A. laibachii, A. loubieri* and *A. porosum* [70,71,72]. *Apiotrichum curvatum* is an oleaginous yeast, which has the ability to accumulate up to 60% of its cellular dry weight as intracellular lipids. When depleted in carbon, cells resorted to endogenous lipids and carbohydrates as carbon and energy sources; the intracellular levels of lipids and carbohydrates decreased by 31 and 26%, respectively [72]. Similarly, *Apiotrichum porosum* (formerly *Trichosporon porosum*) was observed to produce simultaneous single cell oil (SCO) and gluconic acid (GA) in a bioreactor fermentation on glucose, with yields of 17.0 g/L SCO and 12 g/L GA [73].

Regarding *Cutaneotrichosporon* spp. and *Trichosporon* spp., there are articles reporting the ability of these yeasts to accumulate lipids [33,41,74]. The yeast *Cutaneotrichosporon oleaginosum* (formerly *Trichosporon oleaginosus*) was cultured in molasses with 10% sweet potato hydrolysate by Shen et al. [75]. Similarly, Grubišić et al. [4] cultivated the yeast *Cutaneotrichosporon oleaginosum* (formerly *Trichosporon oleaginosus*) in the hydrolysate of alkaline-pretreated corn cobs. They evaluated different process configurations, including separate hydrolysis and fermentation (SHF) with cellulase recycling, and simultaneous scarification and fermentation (SSF) in batch and fed-batch strategy. In fed-batch SSF fed with 2.5% substrate, the highest lipid concentration of 26.74 g L^−1^ was reached at low enzyme loading. In another study conducted by Santek et al. [50], they also evaluated the yeast *Cutaneotrichosporon oleaginosum* (formerly *Trichosporon oleaginosus*) cultured in a lignocellulosic hydrolysate. On the other hand, Kamal et al. [76] utilized hydrolysates from the microalga *Arthrospira platensis* that were converted into lipids by the oleaginous yeast *Cutaneotrichosporon curvatum* ATCC 20509 (formerly *Apiotrichum curvatum*). The results indicated that hydrothermal pretreatment at 120 °C followed by enzymatic hydrolysis led to the recovery of up to 97% of total reducing sugar from the microalgal biomass. While the as-prepared hydrolysates were ineffective for lipid production due to their nitrogen-rich nature, a high lipid content of 64.5% and a lipid yield of 0.233 g/g of dry weight were obtained upon phosphate removal with calcium hydroxide. With regard to the composition of the unsaturated fatty acid profile of strains PP1 and PR27, they indicated 46.53% and 28.55% of oleic acid C18:1 (cis9), respectively. In PUFA, strain PR27 had the highest accumulation of linoleic acid (cis-9 cis-12) C18:2 with 7.25%, PP1 with 2.5% and RHO with 6.37%. Strain PR27 contains C16:0 (45.9%), C18:0 (10.69%) and C18:1 (28.5). In turn, the composition of saturated fatty acids in cocoa butter (CB) contains C16:0 (24.1-25.8%), C18:0 (33.3-37.6%) and C18: 1 (32.7–36.5%). Although there is a similarity in the high content of total saturated fatty acids, there are considerable differences in the lipid profile (C 16:0). Previous studies conducted on the *Trichosporon* spp. strain have identified this particularity [45,47]. Hu et al. [46] investigated the accumulation of lipids in *Trichosporon* sp. obtaining a fatty acid profile of C16:0 (48.6%), C18:0 (19.2%) and C18:1(31.7%), of which 63.4% corresponded to saturated fatty acids (SAFA). These values are very similar to those obtained in strain *A. dulcitum* (formerly *Trichosporon dulcitum*) PR27 (with 65.2% SAFA). In the study conducted by Wei et al. [48], they cultivated six different yeast strains, including *Saccharomyces cerevisiae* and five oleaginous yeast strains. Under the same growth conditions, it was found that TAGs were the main lipids in all six yeast strains and that *Cutaneotrichosporon oleaginosum* (formerly *T. oleaginosus*) was able to produce more TAGs than the other five yeast strains. In general, yeasts contain mainly FA with 16 or 18 carbon atoms, which are either saturated (20%) or monounsaturated (80%) [77]. One of the important aspects to consider in the culture of oleaginous microorganisms is that, depending on the stage of the cell cycle, there is a variation in the lipid profiles, so the study of growth kinetics has a very important role in determining the best culture conditions and harvesting time [17]. Similarly, the use of industrial residues for the culture of microorganisms, such as yeasts, has been one of the alternatives found to try to reduce the production costs of primary or secondary metabolites generated by these unicellular microorganisms [34].

## 5. Conclusions

Depending on their cell yields and fatty acid profiles, yeasts are presented as a potential source of fatty acids. The present work shows that the native yeasts *Debaryomyces* sp. PP1 and *Apiotrichium dulcitum* PR27 are promising strains for the production of microbial oils and can be a useful alternative source to the conventional vegetable oils currently used. Some of the industrial sectors that use vegetable oils in their production processes include the aquaculture and bioenergy (biodiesel) industries. Therefore, the yeasts analyzed in this study may serve as potential sources of oils for these industries.

## Figures and Tables

**Table 1:** Characterization of the IRV.

Parameter	Quantity *
Water	94.1 ± 3 g/L
Total soluble solids	10° ± 0.5° BRIX
Density	1.0047 ± 0.2 g/mL
pH	6.6 ± 0.2
Total protein	2.6 ± 0.3%
Fat matter	0.004 ± 0%
Soluble carbohydrates	8 ± 0.9%
Glucose	1.8 ± 0.2%
Fructose	1.8 ± 0.2%

* Mean ± standard deviation, corresponding to three determinations.

**Table 2:** Biomass production and lipid accumulation in PR27 and PP1 strains cultured for 6 days under different physicochemical conditions.

Factor	Level	Biomass g/L		Lipid Productivity (g L^−1^ Day)		Lipid Content (g/L Lipids)	
		PR27	PP1	PR27	PP1	PR27	PP1
T°C	25	5.21 ± 0.37 *	7.52 ± 0.86 *	0.47 ± 0.11	0.31 ± 0.09	2.83 ± 0.65	1.87 ± 0.49
	30	4.28 ± 0.73	6.35 ± 0.12	0.41 ± 0.17	0.32 ± 0.02	2.417 ± 1.07	1.975 ± 0.97
	37	4.80 ± 0.85	6.03 ± 0.40	0.116 ± 0.10	0.1695 ± 0.02	0.699 ± 0.06	1.017 ± 0.13
pH	4	5.783 ± 0.85	13.86 ± 1.12 *	0.113 ± 0.01	0.286 ± 0.14	0.68 ± 0.06	1.72 ± 0.86
	5.5	7.64 ± 1.29 *	6.49 ± 0.22	0.176 ± 0.02	0.243 ± 0.09	1.056 ± 0.12	1.459 ± 0.59
	7	4.32 ± 0.67	6.53 ± 0.52	0.43 ± 0.12	0.328 ± 0.09	2.63 ± 0.69	1.97 ± 0.58
C/N ratio	20	7.3 ± 0.65	6.03 ± 0.56	0.33 ± 0.13	0.295 ± 0.08	2.03 ± 0.78	1.77 ± 0.50
	50	11.46 ± 0.71	15.83 ± 0.57 *	0.34 ± 0.14	0.38 ± 0.13	2.04 ± 0.69	2.29 ± 0.78
	80	14.8 ± 1.51 *	14.8 ± 1.52	0.65 ± 0.36	0.76 ± 0.02	3.981 ± 0.22	4.614 ± 0.14

* Data represent mean ± standard deviation of three independent experiments.

**Table 3:** Pearson correlation for independent variables and evaluated parameters of strains PP1 and PR27.

Parameter	PP1 Strain				PR27 Strain			
	Biomass		Lipids		Biomass		Lipids	
Temperature	−0.301	*	−0.379		−0.413	*	−0.406	*
C:N ratio	0.876	*	0.833	*	0.895	*	0.655	*
pH	0.033		0.271		0.105		0.586	*

(*) indicate significance for pairwise correlations (*p* < 0.05).

**Table 4:** Growth rate, cell yield and lipid productivity after 144 h of culture.

Yeast	Dry Weight g/L	Y_X_ *	** Lipid Productivity (g L^−1^ d^−1^)	µ (h^−1^)
PP1	14.9 ± 1.52	0.11	0.69	0.14
PR27	11.3 ± 1.71	0.07	0.57	0.12
RHO	16.7 ± 1.11	0.12	0.70	0.19

* Y_X_ = Cell yield (gr. of yeast produced/g. of glucose consumed), ** lipid productivity = grams of yeast per liter of vinasse per day.

## Data Availability

Not applicable.

## Supplementary Materials

The following supporting information can be downloaded at: https://www.mdpi.com/article/10.3390/microorganisms11102516/s1, File S1: Lipids content and Fatty acid profile (mg/g of biomass, % of total fatty acid) determined for each strain evaluated.

## References

[1] Karayannis D., Papanikolaou S., Vatistas C., Paris C., Chevalot I. (2023). Yeast Lipid Produced through Glycerol Conversions and Its Use for Enzymatic Synthesis of Amino Acid-Based Biosurfactants. Int. J. Mol. Sci..

[2] Fabiszewska A., Paplińska-Goryca M., Misiukiewicz-Stępień P., Wołoszynowska M., Nowak D., Zieniuk B. (2022). Expression Profile of Selected Genes Involved in Storage Lipid Synthesis in a Model Oleaginous Yeast Species *Yarrowia lipolytica*. Int. J. Mol. Sci..

[3] Gemperlein K., Dietrich D., Kohlstedt M., Zipf G., Bernauer H.S., Wittmann C., Wenzel S.C., Müller R. (2019). Polyunsaturated fatty acid production by Yarrowia lipolytica employing designed myxobacterial PUFA synthases. Nat. Commun..

[4] Grubišić M., Mihajlovski K., Gruičić A.M., Beluhan S., Šantek B., Ivančić Šantek M. (2021). Strategies for Improvement of Lipid Production by Yeast *Trichosporon oleaginosus* from Lignocellulosic Biomass. J. Fungi.

[5] Jach M.E., Malm A. (2022). *Yarrowia lipolytica* as an alternative and valuable source of nutritional and bioactive compounds for humans. Molecules.

[6] Sirakov I., Velichkova K., Stoyanova S., Staykov Y. (2015). The importance of microalgae for aquaculture industry. Review. Int. J. Fish. Aquat. Stud..

[7] Tacon P., Auclair E. (2010). Yeast in aquaculture from nutrition to well-being. Aqua Cult. Asia Pac..

[8] Sahlmann C., Djordjevic B., Lagos L., Mydland L.T., Morales-Lange B., Hansen J.Ø., Øverland M. (2019). Yeast as a protein source during smoltification of Atlantic salmon (*Salmo salar* L.), enhances performance and modulates health. Aquaculture.

[9] Mekonnen M.M., Hoekstra A.Y. (2014). Water footprint benchmarks for crop production: A first global assessment. Ecol. Indic..

[10] Agboola J.O., Mensah D.D., Hansen J.Ø., Lapeña D., Mydland L.T., Arntzen M.Ø., Horn S.J., Øyås O., Press C.M., Øverland M. (2022). Effects of Yeast Species and Processing on Intestinal Health and Transcriptomic Profiles of Atlantic Salmon (Salmo salar) Fed Soybean Meal-Based Diets in Seawater. Int. J. Mol. Sci..

[11] Patel A., Matsakas L., Sartaj K., Chandra R. (2020). Extraction of lipids from algae using supercritical carbon dioxide. Green Sustainable Process for Chemical and Environmental Engineering and Science.

[12] Sprague M., Betancor M.B., Tocher D.R. (2017). Microbial and genetically engineered oils as replacements for fish oil in aquaculture feeds. Biotechnol. Lett..

[13] Rajak R.C., Rajlakshmi, Saravanabhupathy S., Banerjee R. (2022). Chapter 9 Microbial lipids production by oleaginous yeasts. Biomass Biofuels, Biochemicals.

[14] Karamerou E. (2019). Colin Webb, Cultivation modes for microbial oil production using oleaginous yeasts—A review. Biochem. Eng. J..

[15] Morales-Palomo S., Tomás-Pejó E., González-Fernández C. (2023). Phosphate limitation as crucial factor to enhance yeast lipid production from short-chain fatty acids. Microb. Biotechnol..

[16] Poorinmohammad N., Fu J., Wabeke B., Kerkhoven E.J. (2022). Validated Growth Rate-Dependent Regulation of Lipid Metabolism in *Yarrowia lipolytica*. Int. J. Mol. Sci..

[17] Arous F., Triantaphyllidou I., Mechichi T., Azabou S., Nasri M., Aggelis G. (2015). Lipid accumulation in the new oleaginous yeast *Debaryomyces etchellsii* correlates with ascosporogenesis. Biomass Bioenergy.

[18] Amaretti A., Raimondi S., Sala M., Roncaglia L., De Lucia M., Leonardi A., Rossi M. (2010). Single cell oils of the cold-adapted oleaginous yeast *Rhodotorula glacialis* DBVPG 4785. Microb. Cell Fact..

[19] Papanikolaou S., Aggelis G. (2002). Lipid productionby *Yarrowia lipolytica growing* on industrial glycerol in a single-stage continuous culture. Bioresour. Technol..

[20] Rakicka M., Lazar Z., Dulermo T., Fickers P., Nicaud J.M. (2015). Lipid production by the oleaginous yeast *Yarrowia lipolytica* using industrial by-products under different culture conditions. Biotechnol Biofuels..

[21] Tanimura A., Takashima M., Sugita T., Endoh R., Kikukawa M., Yamaguchi S., Sakuradani E., Ogawa J., Shima J. (2014). Selection of oleaginous yeasts with high lipid productivity for practical biodiesel production. Bioresour. Technol..

[22] Wen Z., Zhang S., Odoh C.K., Jin M., Zhao Z.K. (2020). *Rhodosporidium toruloides*—A potential red yeast chassis for lipids and beyond. FEMS Yeast Res..

[23] Beopoulos A., Haddouche R., Kabran P., Dulermo T., Chardot T., Nicaud J.M. (2012). Identification and characterization of DGA2, an acyltransferase of the DGAT1 acyl-CoA: Diacylglycerol acyltransferase family in the oleaginous yeast *Yarrowia lipolytica*. New insights into the storage lipid metabolism of oleaginous yeasts. Appl. Microbiol. Biotechnol..

[24] Evans C.T., Ratledge C. (1984). Influence of nitrogen metabolism on lipid accumulation by *Rhodosporidium toruloides* CBS 14. Microbiology.

[25] Kot A.M., Błażejak S., Kieliszek M., Gientka I., Bryś J., Reczek L., Pobiega K. (2019). Effect of exogenous stress factors on the biosynthesis of carotenoids and lipids by *Rhodotorula* yeast strains in media containing agro-industrial waste. World J. Microbiol. Biotechnol..

[26] Díaz-Vázquez D., Orozco-Nunnelly D.A., Yebra-Montes C., Senés-Guerrero C., Gradilla-Hernández M.S. (2022). Using yeast cultures to valorize tequila vinasse waste: An example of a circular bioeconomy approach in the agro-industrial sector. Biomass Bioenergy.

[27] Gientka I., Wirkowska-Wojdyła M., Ostrowska-Ligęza E., Janowicz M., Reczek L., Synowiec A., Błażejak S. (2022). Enhancing Red Yeast Biomass Yield and Lipid Biosynthesis by Using Waste Nitrogen Source by Glucose Fed-Batch at Low Temperature. Microorganisms.

[28] Younes S., Bracharz F., Awad D., Qoura F., Mehlmer N., Brueck T. (2020). Microbial lipid production by oleaginous yeasts grown on Scenedesmus obtusiusculus microalgae biomass hydrolysate. Bioprocess Biosyst. Eng..

[29] Muñoz-Tamayo R., Aceves-Lara C., Bideaux C. (2014). Optimization of lipid production by oleaginous yeast in continuous culture. IFAC Proc. Vol..

[30] Soto-Sánchez O., Hidalgo P., González A., Oliveira P.E., Hernández Arias A.J., Dantagnan P. (2023). Microalgae as Raw Materials for Aquafeeds: Growth Kinetics and Improvement Strategies of Polyunsaturated Fatty Acids Production. Aquac. Nutr..

[31] Ledesma-Amaro R., Nicaud J.M. (2016). *Yarrowia lipolytica* as a biotechnological chassis to produce usual and unusual fatty acids. Prog. Lipid Res..

[32] Dyaa A., Soliman H., Abdelrazak A., Samra B.N., Khojah E., Ahmed A.F., El-Esawi M.A., Elsayed A. (2022). Optimization of Carotenoids Production from Rhodotorula sp. Strain ATL72 for Enhancing Its Biotechnological Applications. J. Fungi.

[33] Mota M.N., Múgica P., Sá-Correia I. (2022). Exploring Yeast Diversity to Produce Lipid-Based Biofuels from Agro-Forestry and Industrial Organic Residues. J. Fungi.

[34] Donzella S., Serra I., Fumagalli A., Pellegrino L., Mosconi G., Scalzo R.L., Compagno C. (2022). Recycling industrial food wastes for lipid production by oleaginous yeasts *Rhodosporidiobolus azoricus* and *Cutaneotrichosporon oleaginosum*. Biotechnol. Biofuels Bioprod..

[35] Chang Y., Chang K., Hsu C., Chuang L., Chen C., Huang F., Jang H. (2013). A comparative study on batch and fed-batch cultures of oleaginous yeast *Cryptococcus* sp. in glucose-based media and corncob hydrolysate for microbial oil production. Fuel.

[36] Acevedo F., Gentina J.C., Illanes A. (2002). Fundamentos de Ingeniería Bioquímica, 347.

[37] Zhang L., Lim E.Y., Loh K.-C., Dai Y., Tong Y.W. (2021). Two-Stage Fermentation of *Lipomyces starkeyi* for Production of Microbial Lipids and Biodiesel. Microorganisms.

[38] Poontawee R., Yongmanitchai W., Limtong S. (2018). Lipid production from a mixture of sugarcane top hydrolysate and biodiesel-derived crude glycerol by the oleaginous red yeast, *Rhodosporidiobolus fluvialis*. Process Biochem..

[39] Lapeña D., Olsen P.M., Arntzen M.Ø., Kosa G., Passoth V., Eijsink V.G., Horn S.J. (2020). Spruce sugars and poultry hydrolysate as growth medium in repeated fed-batch fermentation processes for production of yeast biomass. Bioprocess Biosyst. Eng..

[40] Brandenburg J., Blomqvist J., Shapaval V., Kohler A., Sampels S., Sandgren M., Passoth V. (2021). Oleaginous yeasts respond differently to carbon sources present in lignocellulose hydrolysate. Biotechnol. Biofuels.

[41] Gorte O., Kugel M., Ochsenreither K. (2020). Optimization of carbon source efficiency for lipid production with the oleaginous yeast *Saitozyma podzolica* DSM 27192 applying automated continuous feeding. Biotechnol. Biofuels.

[42] Lin J., Shen H., Tan H., Zhao X., Wu S., Hu C., Zhao Z.K. (2011). Lipid production by *Lipomyces starkeyi* cells in glucose solution without auxiliary nutrients. J. Biotechnol..

[43] Tang W., Zhang S., Wang Q., Tan H., Zhao Z.K. (2012). The isocitrate dehydrogenase gene of oleaginous yeast Lipomyces starkeyi is linked to lipid accumulation. Can. J. Microbiol..

[44] Xue Z., Sharpe P.L., Hong S.P., Yadav N.S., Xie D., Short D.R., Damude H.G., Rupert R.A., Seip J.E., Wang J. (2013). Production of omega-3 eicosapentaenoic acid by metabolic engineering of *Yarrowia lipolytica*. Nat. Biotechnol..

[45] Wang M., Wei Y., Ji B., Nielsen J. (2020). Advances in Metabolic Engineering of *Saccharomyces cerevisiae* for Cocoa Butter Equivalent Production. Front. Bioeng. Biotechnol..

[46] Hu C., Wu S., Wang Q., Jin G., Shen H., Zhao Z.K. (2011). Simultaneous utilization of glucose and xylose for lipid production by *trichosporon cutaneum*. Biotechnol. Biofuels.

[47] Konzock O., Matsushita Y., Zaghen S., Sako A., Norbeck J. (2022). Altering the fatty acid profile of *Yarrowia lipolytica* to mimic cocoa butter by genetic engineering of desaturases. Microb. Cell Fact..

[48] Wei Y., Siewers V., Nielsen J. (2017). Cocoa butter-like lipid production ability of non-oleaginous and oleaginous yeasts under nitrogen-limited culture conditions. Appl. Microbiol. Biotechnol..

[49] Díaz P.E., Aranda C., Martínez O., Godoy R., Gonzales A., Valenzuela E. (2017). Characterization of yeast in hapludands soil with biotechnological potential. J. Soil Sci. Plant Nutr..

[50] Adel A., El-Baz A., Shetaia Y., Sorour N.M. (2021). Biosynthesis of polyunsaturated fatty acids by two newly cold-adapted Egyptian marine yeast. 3 Biotech.

[51] Sluiter A., Hames B., Ruiz R., Scarlata C., Sluiter J., Templeton D. (2006). Determination of Sugars, Byprod-UCTS, and Degradation Products in Liquid Fraction Process Samples.

[52] Folch J., Lees M., Sloane Stanley G.H. (1957). A simple method for the isolation and purification of total lipids from animal tissues. J. Biol. Chem..

[53] Maza D.D., Viñarta S.C., Su Y., Guillamón J.M., Aybar M.J. (2020). Growth and lipid production of R C/N R4, in comparison to other oleaginous yeasts. J. Biotechnol..

[54] Poontawee R., Limtong S. (2020). Feeding Strategies of Two-Stage Fed-Batch Cultivation Processes for Micro-bial Lipid Production from Sugarcane Top Hydrolysate and Crude Glycerol by the Oleaginous Red Yeast *Rhodosporidiobolus fluvialis*. Microorganisms.

[55] Guerreiro F., Constantino A., Lima-Costa E., Raposo S. (2018). A new combined approach to improved lipid production using a strictly aerobic and oleaginous yeast. Eng. Life Sci..

[56] Morrison W.R., Smith L.M. (1964). Preparation of fatty acid methyl esters and dimethylacetals from lipids with boron fluoride-methanol. J. Lipid Res..

[57] Vu D., Groenewald M., Szöke S., Cardinali G., Eberhardt U., Stielow B., de Vries M., Verkleij G.J.M., Crous P.W., Boekhout T. (2016). DNA barcoding analysis of more than 9000 yeast isolates contributes to quantitative thresholds for yeast species and genera delimitation. Stud. Mycol..

[58] Tamura K., Stecher G., Kumar S. (2021). MEGA11: Molecular Evolutionary Genetics Analysis Version 11. Mol. Biol. Evol..

[59] Groenewald M., Daniel H.-M., Robert V., Poot G.A., Smith M.T. (2008). Polyphasic re-examination of *Debaryomyces hansenii* strains and reinstatement of *D. hansenii*, *D. fabryi* and *D. subglobosus*. Persoonia.

[60] Silva C.F., Arcuri S.L., Campos C.R., Vilela D.M., Alves J.G., Schwan R.F. (2011). Using the residue of spirit production and bio-ethanol for protein production by yeasts. Waste Manag..

[61] García A., Rojas C. (2006). Posibilidades de Uso de la Vinaza en la Agricultura de Acuerdo con su Modo de Acción en los Suelos. www.tecnicana.org/pdf/2006/tec_v10_no17_2006_p3-13.pdf.

[62] Fernandes B.S., Vieira J.P.F., Contesini F.J., Mantelatto P.E., Zaiat M., Pradella J.G.D.C. (2017). High value added lipids produced by microorganisms: A potential use of sugarcane vinasse. Crit. Rev. Biotechnol..

[63] Ayadi I., Belghith H., Gargouri A., Guerfali M. (2019). Utilization of Wheat Bran Acid Hydrolysate by *Rhodotorula mucilaginosa* Y-MG1 for Microbial Lipid Production as Feedstock for Biodiesel Synthesis. BioMed Res. Int..

[64] Yu X., Zheng Y., Dorgan K.M., Chen S. (2011). Oil production by oleaginous yeasts using the hydrolysate from pretreatment of wheat straw with dilute sulfuric acid. Bioresour. Technol..

[65] Patel A., Sartaj K., Arora N., Pruthi V., Pruthi P.A. (2017). Biodegradation of phenol via meta cleavage pathway triggers de novo TAG biosynthesis pathway in oleaginous yeast. J. Hazard. Mater..

[66] Takashima M., Kurakado S., Cho O., Kikuchi K., Sugiyama J., Sugita T. (2020). Description of four *Apiotrichum* and two *Cutaneotrichosporon* species isolated from guano samples from bat-infested caves in Japan. Int. J. Syst. Evol. Microbiol..

[67] Li A.-H., Yuan F.-X., Groenewald M., Bensch K., Yurkov A.M., Li K., Han P.-J., Guo L.-D., Aime M.C., Sampaio J. (2020). Diversity and phylogeny of basidiomycetous yeasts from plant leaves and soil: Proposal of two new orders, three new families, eight new genera and one hundred and seven new species. Stud. Mycol..

[68] Li Q., Xiao W., Wu P., Zhang T., Xiang P., Wu Q., Zou L., Gui M. (2023). The first two mitochondrial genomes from Apiotrichum reveal mitochondrial evolution and different taxonomic assignment of Trichosporonales. IMA Fungus.

[69] Liu X.Z., Wang G.M., Göker M., Groenewald M., Kachalkin A.V., Lumbsch H.T., Millanes A.M., Wedin M., Yurkov A.M., Bai F.Y. (2015). Towards an integrated phylogenetic classification of the Tremellomycetes. Stud. Mycol..

[70] Aliyu H., Gorte O., de Maayer P., Neumann A., Ochsenreither K. (2020). Genomic insights into the lifestyles, functional capacities and oleagenicity of members of the fungal family Trichosporonaceae. Sci. Rep..

[71] Qian X., Zhou X., Chen L., Zhang X., Xin F., Dong W., Zhang W., Ochsenreither K., Jiang M. (2020). Bioconversion of volatile fatty acids into lipids by the oleaginous yeast Apiotrichum porosum DSM27194. Fuel.

[72] Park W.-S., Murphy P.A., Glatz B.A. (1990). Lipid metabolism and cell composition of the oleaginous yeast *Apiotrichum curvatum* grown at different carbon to nitrogen ratios. Can. J. Microbiol..

[73] Gorte O., Aliyu H., Neumann A., Ochsenreither K. (2019). Draft Genome Sequence of the Oleaginous Yeast *Apiotrichum porosum* (syn. *Trichosporon porosum*) DSM 27194. J. Genom..

[74] Šantek M.I., Lisičar J., Mušak L., Špoljarić I.V., Beluhan S., Šantek B. (2018). Lipid Production by Yeast *Trichosporon oleaginosus* on the Enzymatic Hydrolysate of Alkaline Pre-treated Corn Cobs for Biodiesel Production. Energy Fuels.

[75] Shen Q., Lin H., Wang Q., Fan X., Yang Y., Zhao Y. (2015). Sweetpotato vines hydrolysate promotes single cell oils production of *Trichosporon fermentans* in high-density molasses fermentation. Bioresour. Technol..

[76] Kamal R., Huang Q., Li Q., Chu Y., Yu X., Limtong S., Xue S., Zhao Z.K. (2021). Conversion of *Arthrospira platensis* Biomass into Microbial Lipids by the Oleaginous Yeast *Cryptococcus curvatus*. ACS Sustain. Chem. Eng..

[77] Shaigani P., Awad D., Redai V., Fuchs M., Haack M., Mehlmer N., Brueck T. (2021). Oleaginous yeasts- substrate preference and lipid productivity: A view on the performance of microbial lipid producers. Microb. Cell Fact..

