# Supplementary material for: Lipid Production from Native Oleaginous Yeasts Isolated from Southern Chilean Soil Cultivated in Industrial Vinasse Residues
Source: Microorganisms. 2023 Oct 9;11(10):2516. doi: 10.3390/microorganisms11102516 (PMC10609240; doi:10.3390/microorganisms11102516)
Supplement: Supplementary file 1 [file microorganisms-11-02516-s001.zip › microorganisms-2467085-supplementary file s1.pdf]

File S1. Lipids content and Fatty acid profile (mg/g of biomass, % of total fatty acid) determined for each strain evaluated.

		PR27	PP1	PR27
		mg/g ± SD (%)	mg/g ± SD (%)	mg/g ± SD (%)
**Total lipids**		302 ± 2.8 (28.0)	289 ± 24.1 (28.9)	258 ± 8.8 (25.8)
**Saturated fatty acids (SAFA)**				
C4:0	*Butanoic acid*	–	–	–
C6:0	*Caproic acid*	–	–	–
C8:0	*Caprylic acid*	–	–	–
C10:0	*Capric Acid*	–	–	–
C12:0	*Lauric Acid*	2.7 ± 3.9 (1.8)	–	1.06 ± 0.0 (0.4)
C13:0	*Tridecanoic acid*	–	–	–
C14:0	*Myristic acid*	20.2 ± 2.7 (6.7)	4.38 ± 1.8 (1.6)	12.01 ± 2.7 (4.6)
C15:0	*Pentadecanoic Acid*	–	–	2.03 ± 1.3 (0.8)
C16:0	*Palmitic acid*	139.0 ± 34.4 (46.0)	91.23 ± 5.3 (31.6)	89.63 ± 5.3 (34.8)
C17:0	*Heptadecanoic acid*	–	7.87 ± 4.2 (2.7)	6.29 ± 0.6 (2.4)
C18:0	*Stearic acid*	32.3 ± 0.3 (10.7)	32.58 ± 0.1 (11.3)	15.02 ± 0.9 (5.8)
C20:0	*Arachidic Acid*	–	–	–
C21:0	*Heneicosanoic acid*	–	–	–
**Monounsaturated fatty acids (MUFA)**				
C14:1	*Myristoleic acid*	–	–	–
C15:1	*Cis-10-Pentadecenoic acid*	–	–	–
C16:1t	*Palmitelaidic Acid*	–	–	1.58 ± 0.0 (0.6)
C16:1	*Palmitoleic acid*	–	6.95 ± 1.5 (2.4)	7.19 ± 4.3 (2.8)
C16:1	*Palmitoleic acid*	–	–	2.53 ± 0.0 (1.0)
C17:1	*Cis-10-Heptadecenoic acid*	–	8.44 ± 0.00 (2.8)	8.55 ± 0.0 (3.4)
C18:1t	*trans-Vaccenic acid*	–	–	–
C18:1n9t	*Elaidic Acid*	–	–	11.40 ± 0.0 (4.3)
C18:1n9c	*Oleic acid*	86.0 ± 22.9 (28.5)	134.34 ± 11 (46.5)	79.13 ± 17 (30.8)
C18:1	*Vaccenic acid*	–	–	–
C20:1n9	*Cis-11-Eicosenoic Acid*	–	–	–
C22:1n9	Erucic acid	–	–	–
**Polyunsaturated fatty acids (PUFA)**				
C18:2n6t	*Linolelaidic Acid*	–	–	–
C18:2n6c	*Linoleic acid (cis-9 cis-12)*	21.8 ± 15.1 (7.3)	7.18 ± 1.5 (2.5)	16.51 ± 5.0 (6.4)
C18:2	*Linoleic acid (cis-10 cis-12)*	–	–	3.96 ± 0.0 (1.5)
C18:2	*Rumenic acid (cis-9, trans-11)*	–	–	–
C18:3n3	*Linolenic Acid*	–	–	9.06 ± 0.0 (3.4)
C18:3n6	*g-linolenic acid*	–	–	–
C20:2	*Eicosadienoic Acid*	–	–	21.81 ± 0.0 (8.3)
C20:3n3	*Eicosatrienoic Acid*	–	–	–
C20:4n6	*Arachidonic Acid*	–	–	–
C20:5n3	*Eicosapentaenoic Acid*	–	–	–
C22:2	*Docosadienoic acid*	–	–	–
C22:4	*Docosatetraenoic acid*	–	–	–
C22:5n6	*Docosapentaenoic acid*	–	–	–
C22:6n3	*Docosahexaenoic acid*	–	–	–
	**∑ SAFA**	194.3 ± 40.7 (64.3)	136.1 ± 7.6 (47.2)	125.5 ± 4.7 (48.8)
	**∑ MUFA**	86.0 ± 22.9 (28.5)	145.5 ± 18.0 (50.3)	98.4 ± 16.1 (38.3)
	**∑ PUFA**	21.8 ± 15.1 (7.3)	7.2 ± 1.5 (2.5)	33.9 ± 29.7 (13.0)

∑SAFA: Total saturated fatty acids.
∑MUFA: Total monounsaturated fatty acids.
∑PUFA: Total polyunsaturated fatty acids.
– : Fatty acid not detected.
* The values correspond to means ± SD n=2 of each strain, in parentheses the percentage with respect to total lipids.
